# TRIM29 is Critical for Bladder Cancer Progression and Modulates the Tumor-Immune Niche

**DOI:** 10.21203/rs.3.rs-9214925/v1

**Published:** 2026-05-20

**Authors:** Phillip Palmbos, Alan Kelleher, Marian Henderson, Luke Broses, Yin Wang, Nicole Jerome, Christopher Patsalis, Armand Bankhead, Kathleen Day, Aaron Udager, Yu Leo Lei, Barbara Foster, Wendy Huss, Mark Day

**Affiliations:** University of Michigan Health System; University of Michigan Health System; University of Michigan Health System; University of Michigan Health System; Mayo Clinic; University of Michigan Health System; University of Michigan Health System; University of Michigan Health System; University of Michigan Health System; University of Michigan; Roswell Park Cancer Institute; Roswell Park Cancer Institute

**Keywords:** Bladder cancer, TRIM29, Immune Microenvironment

## Abstract

TRIM29 (Tripartite Motif-Containing Protein 29), also known as ATDC (Ataxia-Telangiectasia Group D-Complementing Protein), is an E3 ubiquitin ligase frequently upregulated in muscle-invasive bladder cancer (MIBC). We have previously shown that TRIM29 overexpression in a genetically engineered mouse model (GEMM) is sufficient to drive development of MIBC. We have also shown that TRIM29 is enriched in basal bladder cancer subtypes and is part of a TP63-regulated program that promotes migration and invasive progression. Prior studies suggest that Keratin 5/14 (K5+/K14+) expressing basal-stem progenitor cells in the urothelium are the cells of origin for MIBC in mice. However, whether Trim29 expression in these basal urothelial cells is required for MIBC development has not been established. In this study, we find that Trim29 expression in K5+ basal cells is linked to the inflammatory response and is critical for bladder tumor formation and invasive progression. We show that urothelial TRIM29 expression is enhanced during inflammation in humans and under inflammatory and genotoxic stress in mice. Knockout (KO) of Trim29 in the K5+ basal urothelium of a GEMM exposed to a bladder-specific chemical carcinogen blocked MIBC development. Further, urothelial Trim29 KO enriches immune cell recruitment to the bladder and upregulates STING and inflammatory signaling in mouse and human bladder cancers. These data reveal a critical role for TRIM29 in immunomodulation of the bladder tumor microenvironment (TME), which may act in concert with pro-migratory basal gene programs to expedite MIBC development.

## Introduction

Bladder cancer is the 6th most common malignancy in the United States and results in nearly 17,000 deaths per year [1]. The majority of newly diagnosed cases are non-muscle-invasive (NMIBC) and are usually manageable with a combination of transurethral resection (TURBT) and intravesical adjuvant therapy [2]. However, these tumors often recur and can progress to muscle-invasive bladder cancer (MIBC), the most lethal form of the disease. Once tumors acquire the ability to invade the muscularis propria, ~ 50% will metastasize and metastatic disease has a 5-year overall survival (OS) of < 10% [3]. Systemic therapies, including platinum-based chemotherapy, antibody-drug conjugates, and immunotherapy are used to manage metastatic disease, but these treatments are not considered curative. A better understanding of the molecular underpinnings of MIBC development would allow improved prognostics and the development of more effective therapeutic strategies to control progression.

In a normal adult bladder, the urothelium is organized into distinct layers of cells, classified by co-expression of lineage-specific markers [4, 5]. Luminal-facing umbrella cells form the superficial barrier and express terminal differentiation markers, including KRT20 (K20) and uroplakins (UPKs), but lack basal specific markers and TP63 expression [6]. In contrast, basal urothelial cells that line the basement membrane are enriched for KRT5 (K5) and express TP63, serving as a progenitor-like compartment with regenerative potential. Transcriptomic profiles from TCGA and other groups have established that bladder cancer has well-defined basal and luminal molecular subtypes [7–9]. Basal bladder cancers are characterized by the expression of basal markers (KRT14, KRT5, TP63), increased invasiveness, and worse clinical outcomes.

We previously found that Tripartite Motif-Containing Protein 29 (TRIM29), also known as Ataxia-Telangectasia Group D-Complementing Protein (ATDC), is an important driver of basal bladder cancer initiation and invasive progression [10, 11]. TRIM29 is upregulated by TP63 as part of a basal gene program, which is important for bladder tumor progression and metastasis [11]. We have also shown that TRIM29 promotes tumor cell migration and invasion by interacting with KRT14 filaments and stabilizing focal adhesion complexes [12]. Although TRIM29 is linked to basal differentiation in basal/squamous bladder cancer and epithelial cell migration, its expression across the urothelium and in developing tumors has not been well-characterized.

Tumor-associated inflammation is an important hallmark of developing cancers [13]. While acute inflammatory mechanisms are critical for epithelial cell regeneration and tissue repair, chronic insults to the bladder urothelium (e.g. tobacco smoke, aromatic amines in hair dyes, Schistosomiasis infections) are a significant risk factor for bladder tumorigenesis and progression [14–16]. In mice, exposure to the tobacco-related carcinogen, N-butyl-N-(4-hydroxybutyl)-nitrosamine (BBN), induces activation and hyperproliferation of basal stem populations in the urothelium and drives tumorigenesis and progression via sustained genotoxicity and proinflammatory remodeling [17–19].

TRIM29 has also been implicated as a negative regulator of inflammatory signaling through its ability to target key innate immune adaptor proteins for degradation. In myeloid cells, TRIM29 has been shown to limit type-I interferon (IFN) production and proinflammatory cytokine expression via K48-linked ubiquitination and proteasomal degradation of the Stimulator of Interferon Genes (STING), a central mediator of cytosolic DNA sensing [20]. TRIM29 has also been shown to further regulate antiviral and inflammatory pathways by targeting Mitochondrial Antiviral-Signaling Protein (MAVS) and NF-kappa-B Essential Modulator (NEMO), two essential mediators of RIG-I-like receptor and NF-κB signaling [21–23]. Recent reports have shown that in mouse intestinal epithelial cells, TRIM29 targets inflammasome components for proteasomal degradation during viral infection, limiting cytokine production and lymphocyte recruitment [24]. These studies suggest that TRIM29 is broadly associated with inflammatory signaling, acting as a negative regulator of innate immune activation in professional antigen-presenting cells and epithelial cells. However, how TRIM29 modulates innate immune signaling in the bladder epithelium upon urothelial insult and remodeling, and whether this pathway contributes to TRIM29-mediated bladder tumorigenesis was unknown.

We hypothesized that TRIM29 is required for bladder tumor invasive progression and that TRIM29 is associated with suppression of innate immune signaling in the tumor microenvironment (TME). To test this, we generated a urothelial-specific *Trim29* inducible knockout (KO) genetically engineered mouse model (GEMM). In this model, bladder tumorigenesis was driven by chronic exposure to the bladder-specific carcinogen BBN in mouse drinking water. Interestingly, Trim29 expression is dynamically upregulated in BBN-exposed bladder tissue in mice and similarly upregulated in chronic bladder inflammation in humans. We found that *Trim29* KO in the K5-expressing (K5+) basal urothelium significantly impaired tumorigenesis and invasive progression. Moreover, *Trim29* KO resulted in upregulation of STING in the urothelium and was associated with significant remodeling of the immune TME in mice and human bladder cancer. Overall, these findings suggest that TRIM29 is a critical mediator of tumor formation, invasive progression, and suppression of anti-tumor inflammatory signaling in the bladder.

## Materials and Methods

### Human Samples and Tissue Microarray

Deidentified individual human bladder cancer and normal urothelial samples were obtained from Dr. Aaron Udager (University of Michigan, Ann Arbor, MI). Human bladder cancer tissue microarray (TMA), BL2081c, was purchased from www.tissuearray.com, Derwood, MD (formerly US Biomax, Inc). This pathology grade TMA contains 145 cases of urothelial carcinoma, 16 squamous cell carcinoma, 15 adenocarcinoma, 8 each of adjacent normal tissue and bladder tissue, in a single core per case.

### Histology and Immunohistochemistry (IHC)

Formalin-fixed tissues were processed through graded alcohols and cleared with xylene followed by infiltration with molten paraffin using an automated VIP5 or VIP6 tissue processor (TissueTek, Sakura-Americas). Following paraffin embedding using a Histostar Embedding Station (Thermo Scientific), tissues were then sectioned on a RM2255 rotary microtome (Leica Biosystems) at 4 μm thickness, mounted on glass slides, and heated to 60°C for 1 hour. For IHC, following deparaffinization and hydration with xylene and graded alcohols, formalin-fixed, paraffin embedded slides were subjected to heat-induced epitope retrieval (HIER) in a Biocare Decloaking Chamber (pressure cooker) for approximately 40 minutes (maximum temperature of 127°C) in Diva buffer, citrate based, pH 6 (Biocare Medical #DV2004). IHC staining performed on an automated Biocare IntelliPATH FLX immunohistochemical stainer included endogenous peroxidase blocking, Fc Receptor and non-specific background blocking (for F4/80), primary antibody incubation diluted in DaVinci Diluent (Biocare Medical, Cat# PD900), detection using a horseradish peroxidase biotin-free polymer-based system, disclosure with diaminobenzidine (DAB) chromogen (Biocare Medical #IPK5010G80), chromogen enhancement in DAB Sparkle (Biocare Medical #DS830) for 1 minute, nuclear counterstaining with hematoxylin for 5 minutes, dehydrating and clearing through graded alcohols and xylene, and coverslipping with Micromount, using a Leica CV5030 automatic coverslipper (Leica Biosystems). A list of antibodies use for IHC can be found in Supplemental Table 1.

### IHC Quantitation

Quantitation of TRIM29 IHC in human samples was measured using QuPath (Version 0.6.0) [25]. A large representative region of interest (ROI) encompassing either tumor or urothelium (normal and tumor adjacent) was annotated on each core of TMA BL2081c. Mean 3,3′-diaminobenzidine (DAB) staining intensity, a surrogate marker of protein expression, for TRIM29 IHC was then calculated for each case.

### Cell Culture

UM-UC9, SV-HUC1, and UM-UC14 and UM-UC5 wild-type (WT) and *TRIM29* KO cells were cultured in Gibco Dulbecco’s Modified Eagle Medium (DMEM) (Fisher Scientific, Cat # 11–9950973) supplemented with 10% Gibco fetal bovine serum (FBS) (Fisher Scientific, Cat # 26140–079, Lot # 2619962RP) and 1% Gibco Antibiotic-Antimycotic (100X) (Fisher Scientific, Cat # 15–240-062) at 37°C and 5% CO_2_ in Falcon Standard Tissue Culture Dishes (Fisher, Cat # 08–772E). All cell lines were fingerprinted and mycoplasma-tested negative. *TRIM29* KO bladder cancer cell lines were generated as previously described [11].

### TNF-α Stimulation

Human Tumor Necrosis Factor alpha (hTNF-α) was obtained from Cell Signaling Technology (#8902). SV-HUC1 and UM-UC9 cells were exposed to 20 ng/mL of hTNF-α for 0.5, 1, 3 or 6 hours and cells were lysed and examined by western blotting for TRIM29, NF-kB (p65) and phospho-NF-kB (Supplemental Table 2).

### BBN-Induced Bladder Carcinogenesis

To induce bladder tumor development, mice were exposed to 0.05% N-butyl-N-(4-hydroxybutyl)-nitrosamine (BBN, Sigma #B8061) in drinking water *ad libitum* for up to 24 weeks starting at 2–3 months of age. Tamoxifen induction began three weeks prior to the initiation of BBN exposure to allow for Cre-mediated recombination and continued concurrently with BBN for an additional three weeks. After this period, mice were returned to a standard chow diet, and BBN exposure continued uninterrupted for the remainder of the 24-week experiment. Control mice underwent identical handling and dietary conditions. Mice were monitored weekly for weight, general health, and signs of hematuria. At study endpoint, mice were euthanized, and bladders were harvested, weighed, and fixed in 10% neutral buffered formalin for histological evaluation. For monitoring Trim29 expression in a BBN time course, B6 albino mice (B6(Cg)-*Tyr*^*c−2J*^/J; Jackson Laboratory) were administered BBN water for acute (2–4 week) or chronic (12 week) durations, or acute exposure followed by an 18-week recovery period (tap water).

#### Mouse Models and Tamoxifen-Inducible Trim29 Knockout

*Trim29* (*ATDC*) ^flox/flox^ mice were generated as previously described [26]. K5^CreERT2^ mice (FVB.Cg-Tg(KRT5-cre/ERT2)2Ipc/JeldJ) were obtained from Dr. L. Lei at the University of Michigan (Ann Arbor). *Krt5* (K5) *Trim29* inducible knockout mice were generated by mating *Trim29*^flox/flox^ mice with K5^CreERT2^ mice to generate K5^CreERT2^; *Trim29*^*flox/WT*^ mice. Mice heterozygous for K5^CreERT2^ and *Trim29*^*flox*^*were then mated back to homozygous Trim29*^*flox/flox*^ mice to generate K5^CreERT2^; *Trim29*^*flox/flox*^*mice*. Deletion of *Trim29* exons 2–4 in the K5 + basal urothelium was achieved by tamoxifen administration via chow (500mg/kg Tamoxifen, 2016, Teklab Global 16% Protein Rodet Diet, inotiv, Cat# TD.130858) *ad libitum* for six weeks to induce Cre-recombinase activity in K5 + basal urothelial cells. *Trim29* WT littermate control cohorts included: (B) *Trim29*^flox/flox^ (Tamoxifen chow), (C) K5^CreERT2^;*Trim29*^flox/flox^ (regular chow), (D) *Trim29*^flox/flox^ (regular chow). All animal procedures were approved by the University of Michigan Institutional Animal Care and Use Committee (IACUC protocol #: PRO00011234).

### Genotyping

Genotyping for *Trim29* WT, *Trim29*^flox/flox^ and K5^CreERT2^ alleles was performed as previously described [26]. Briefly, mouse tail DNA was prepared using REDExtract-N-AmpTissue PCR Kit (Sigma-Aldrich, Cat# XNAT-1000RXN) and amplified in a BioRad T100 Thermal cycler with 35 cycles of 94°C for 30 s, 55°C for 30 s and 72°C for 30 s. Primers for PCR genotyping were as follows. Primers for genotyping the *Cre* allele were 5′-GAACCTGATGGACATGTTCAGG-3′ (forward), 5′-AGTGCGTTCGAACGCTAGAGCCTGT-3′ (reverse), generating a 320-base-pair (bp) product. Primers for genotyping *Trim29*^flox/flox^ mice were 5’-CCAGCTCTTGCTTGCAACACCAGG-3’ (floxed forward), 5’-TGTTCCTTTTGCAGTGACAGCTCG-3’ (floxed reverse), 5’-CAACGGGTTCTTCTGTTAGTCC-3’ (wild-type reverse), generating a 500 bp *Trim29* WT product and/or a 650 bp *Trim29* floxed product.

### BBN Tumor Staging

H&E slides were deidentified for evaluation by a genitourinary urologist blinded to genotype (A. Udager). Bladders were characterized and staged based on the degree of invasion, cytologic atypia, and squamous or urothelial morphology. Bladder tumor stages were categorized as stromal invasive (T1), muscle-invasive (≥ T2), extensive (tumor burden > 50% of bladder section), and non-invasive (dysplasia/Ta/Carcinoma in situ (CIS)).

### Multiplex Immunofluorescence (mIF) Staining and Slide Scanning

After optimization of the staining parameters for each individual primary antibody, the slides from BBN-exposed bladders were sequentially stained with a mouse specific antibody panel listed in Supplemental Table 3. Staining for TMA BL2081c used a human specific panel (H1) listed in Supplemental Table 4. Staining for individual human bladder cancer samples used human specific panel (H2) listed in Supplemental Table 4. Slides were stained using a Ventana Discovery Ultra stainer. After the primary antibody incubation, the slide was washed with TBST and Ventana Omnimap anti-rabbit (or anti-mouse) HRP was applied. The application of Opal Tyramide Signal Amplification (TSA) resulted in the precipitation of the fluorochrome at the site of the antigen-antibody complex. Subsequent heat induced epitope retrieval using CC2 buffer removed the primary antibody/secondary antibody complex, and the next antibody was applied. Thus, a panel of six fluorochromes was sequentially applied to the tissue. The slides were then mounted with Prolong gold containing DAPI and scanned using the Vectra Polaris automated quantitative pathology imaging system (Akoya Biosciences) at 20X magnification.

### mIF Image Analysis

Digital mIF slide scans from BBN-exposed mouse bladders and human samples were annotated manually with Phenochart software (version 1.0.11 Akoya Biosciences, Inc.). 3–5 Regions of interest (ROIs) on each image were stamped on areas encompassing the tumor/urothelial-stromal interface for both *Trim29* KO bladders (n = 6) and WT bladders (n = 7). ROIs were analyzed using InForm image analysis software (version 2.4.3 Akoya Biosciences, Inc.). Briefly, trainable machine learning algorithms were generated using representative ROIs to segment tumor/urothelial and stromal tissue compartments. DAPI fluorescence was used to segment cells and CD8, CD4 or CD3, and CK fluorescence were used to assist in nuclear splitting. Following cell segmentation, trainable phenotyping algorithms with identical segmentation parameters were generated separately for each expression marker (Trim29, Foxp3) and a single algorithm for lineage markers (CD4, CD8, CK, CD206). Trained phenotyping algorithms were then applied to all mIF digital slide scans in batch and processed data exported. Exported data was merged, consolidated and analyzed using the phenoptrReports application in the phenoptr package (version 0.3.2, Akoya Biosciences, Inc.) in RStudio (version 2024.12.0 + 467). For the BL2081c TMA, each core was annotated using a TMA grid in Phenochart and algorithms for data processing were generated with representative cores as described above. Phenotypes that were assessed by combinatorial marker expression and analyzed are listed in Supplemental Table 5 (mouse) and Supplemental Table 6 (human).

### Gene Knockdowns

SMART pool ON-TARGETplus siRNAs targeting *TRIM29* (L-012409–00-0005) and non-targeting controls (D-001810–10-05) were obtained from Horizon Discovery and transfected into cells using Lipofectamine RNAiMAX transfection reagent (13778100, ThermoFisher Scientific) as previously described [11].

### Immune Stimulation and RT-qPCR

Double-stranded DNA (dsDNA) stimulation of UM-UC5 and UM-UC14 bladder cancer cells (WT or *TRIM29* KO) was adapted from and performed as described previously [27]. Briefly, cells were transfected with 2.5ug poly(dA:dT) (Invivogen # tlrl-patn) or 10ug interferon-stimulatory DNA (ISD Invivogen #tlrl-isdn) using lipofectamine 3000 reagent (Fisher, Cat # L3000150) and cultured for 9 or 16 hours. RNA was extracted using RNeasy Mini Kit and RNase-free DNase Set (Qiagen, Cat # 74104 and 79253), and cDNA was synthesized with iScript cDNA Synthesis Kit (BioRad, Cat # 1708891). RT-qPCR was performed using iTaq Univer SYBR Green SMX (BioRad, Cat # 1725122) in the QuantStudio 5 System apparatus with 40 cycles of 15 seconds at 95°C and 60 seconds at 60°C. All reactions were performed in triplicate. mRNA expression was normalized to endogenous β-actin, and fold change was calculated using the ΔΔCt method relative to lipofectamine treated controls. A list of probes manufactured by Invitrogen are listed in Supplemental Table 7.

### Bru-seq and Transcriptomic Analysis

Nascent RNA profiling was performed by 5-bromouridine (BrU) labeling and Bru-seq, as described previously [28]. Briefly, UM-UC14 cells were incubated with BrU for 30 minutes, followed by RNA extraction, immunoprecipitation, and library preparation. Sequencing was performed on a llumina Hiseq2000. Differential expression analysis was performed and gene set enrichment analysis (GSEA) was performed using Hallmark gene sets (FDR q < 0.05).

### Statistical Analysis

Statistical analyses were performed in RStudio (version 2024.12.0 + 467) or GraphPad Prism (Version 10.2.3). Comparisons between two groups were performed using two-tailed unpaired Student’s t-test or Wilcoxon rank-sum test as appropriate. Tumor stage frequencies were compared using Fisher’s exact test. P-values < 0.05 were considered statistically significant. Statistical comparisons between *TRIM29* WT and KO mRNA expression were performed using one-way ANOVA to determine overall statistical significance and pairwise comparisons were conducted using Bonferroni-corrected t-tests.

### Data Availability

All data and reagents presented in the results were generated by the authors and available on request.

## Results

**Figure 1:**
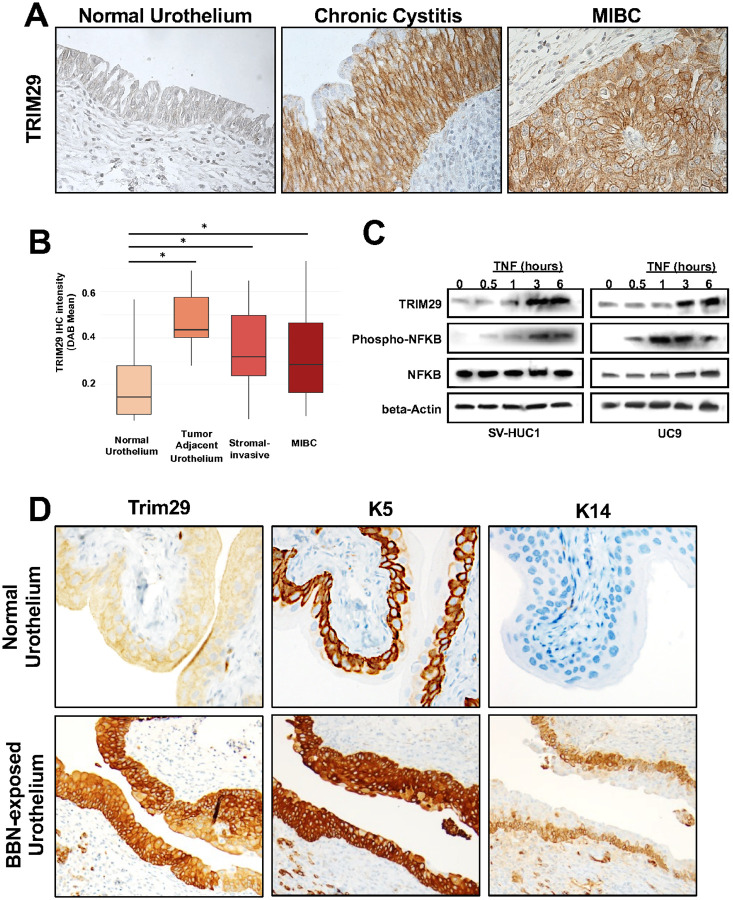
Inflammation increases TRIM29 expression in the Urothelium. (A) TRIM29 expression is increased in the urothelium of chronic cystitis patients and patients with muscle-invasive bladder cancer (MIBC) as evidenced by IHC. (B) TRIM29 IHC DAB mean quantitation of human blader cancer TMA BL2081c. TRIM29 is significantly upregulated in bladder tumors and tumor-adjacent urothelium as compared to normal urothelium without adjacent tumor. Normal (n = 8), Tumor adjacent (n = 6), T1 stromal invasive (n = 49), T2 MIBC (n = 83). Statistical significance was assessed using the Kruskal-Wallis test (p = 0.03) followed by pairwise Dunn’s tests with Benjamini-Hochberg correction for multiple comparisons. Comparisons with FDR-adjusted p < 0.05 were considered significant. (C) Treatment of normal immortalized human urothelium (SV-HUC1) and bladder cancer cell lines (UM-UC9) with TNF-a results in increased TRIM29 expression as well as increased pNF-κB. (D) BBN exposure results in increased Trim29 expression and expansion of K5 and K14 basal urothelial cell populations in mice.

TRIM29 is upregulated in response to inflammatory stress. TRIM29 is a key driver of bladder cancer formation and is upregulated as part of a basal gene program regulated by TP63 [10, 11]. However, the events which drive activation of this basal gene program and upregulate TRIM29 in the bladder were not previously known. Several groups have identified TRIM29 upregulation as a response to inflammatory signaling in immune and epithelial cells [21–24]. In addition, chronic urothelial insult and inflammation either from infection or chemical carcinogens is a known risk factor for development of bladder cancer in humans [29]. To determine if inflammation was a driving event for TRIM29 upregulation in the bladder urothelium, we compared TRIM29 expression in normal human bladder tissue, chronic cystitis and in MIBC. We found that TRIM29 expression was markedly increased in both chronic cystitis and MIBC as assessed by IHC (Fig. 1A). To examine if TRIM29 is persistently elevated in the urothelium in which human bladder tumors arise, we performed IHC for TRIM29 on a progressive bladder cancer TMA and quantitated expression using image analysis software. We found that TRIM29 was persistently elevated in the urothelium adjacent to tumor (n = 6), T1 stromal-invasive bladder cancer *(n = 49)*, and T2 MIBC *(n = 83)* as compared to normal urothelium (n = 8) (Fig. 1B). These results suggested that TRIM29 is upregulated in the inflamed urothelium. To determine if inflammatory signals could upregulate TRIM29 in normal urothelial and bladder cancer cells, we next treated SV-HUC1 (immortalized normal urothelium) and UM-UC9 (bladder cancer) cell lines with TNF-α. We found that treatment with TNF-α induced increased protein levels of TRIM29 in both cell lines (Fig. 1C). These results suggest that TRIM29 upregulation is a response to inflammatory stimuli in the urothelium.

Tobacco exposure is one of the primary drivers of urothelial cancer formation in humans [30]. BBN is a nitrosamine DNA-alkylating carcinogen, whose metabolites mimic the genotoxic, mutagenic and inflammatory effects of tobacco smoke on the human bladder [18, 31]. Mice exposed to 0.05% BBN in drinking water develop CIS, papillary, and basal/squamous histology with both non-invasive and muscle-invasive components after 16–24 weeks [32]. These tumors display similar mutations and molecular signatures to their human counterparts [33]. BBN exposure also induces hyperplasia and inflammatory remodeling of the mouse urothelium [19]. We therefore hypothesized that BBN exposure would drive upregulation of Trim29 in the urothelium of mice. To examine this, we delivered BBN *ad libitum* (0.05% in drinking water) to mice and examined Trim29 and basal marker expression after 24 weeks. As expected, BBN exposure resulted in increased urothelial expression of Trim29 which was accompanied by expansion of K5 + and K14 + basal urothelial and progenitor populations (Fig. 1D). Trim29 upregulation was evident after two weeks of BBN exposure and intensified with chronic 12-week exposure but returned to near-baseline levels following a prolonged recovery period in the absence of BBN (Sup. Figure 1). Taken together, these results suggest that TRIM29 is upregulated in response to inflammation and urothelial injury, and that TRIM29 upregulation is reversible after resolution of inflammatory stress.

**Figure 2:**
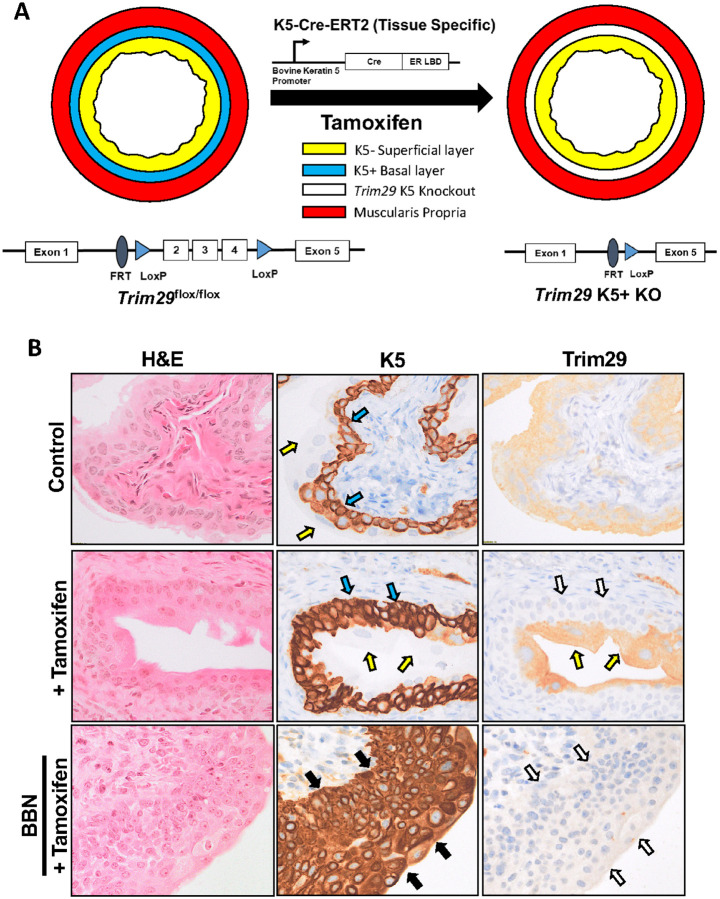
Inducible *Trim29* KO GEMM. (A) Schematic showing K5^CreERT2^ regulated *Trim29* KO Model. Tamoxifen administration results in deletion of exons 2–4 in K5+ basal urothelial cells. (B) Representative H&E and IHC images of model system. Normal (control) urothelium is made up of basal K5+ cells (blue arrows) and K5-negative superficial cells (yellow arrows). Tamoxifen administration results in *Trim29* KO in K5+ basal cells only (middle row panels, white arrows). In BBN-exposed bladders, K5-negative intermediate and umbrella cells are lost resulting in only K5+ basal cells (black arrows) and complete urothelial *Trim29* KO (bottom row panels, white arrows).

Generation of K5 + Basal Cell Trim29 KO GEMM. We have previously shown that TRIM29 is highly expressed in > 70% of human MIBCs and that overexpression of TRIM29 is sufficient to induce the development of MIBC in GEMMs [10]. We have also shown that TRIM29 expression is enriched in basal subtype tumors compared to luminal tumors [11]. This aligns with known transcriptional regulation of basal genes, including *KRT14* and *TRIM29* by TP63, a key driver of the basal phenotype [12]. Others have shown that MIBC develops predominately from K5 + cells which form the basal layer and overpopulate the urothelium following insult (Fig. 1D) [32, 34]. Therefore, we hypothesized that Trim29 expression in K5 + basal cells is required for the formation of MIBC in mice. To examine the role of TRIM29 in basal bladder cancer progression, we generated an inducible *Trim29* KO GEMM using the tamoxifen-inducible bovine Cre-ERT2 system. *Trim29*^flox/flox^ mice were crossed with K5^CreERT2^ mice to generate K5^CreERT2^; Trim29^flox/flox^ mice. Tamoxifen delivery induces Cre recombinase activity which initiates the excision of *Trim29* exons 2–4 exclusively in K5 + basal cells (Fig. 2A). Bladder sections from these GEMMs administered a tamoxifen diet showed no structural differences in urothelial architecture when compared to untreated mice, indicating that basal cell depletion of Trim29 does not upset tissue morphology (Fig. 2B). K5 + cells are readily detected along the normal urothelial basement membrane (Fig. 2B, middle column, blue arrows). Importantly, Trim29 staining was absent in the urothelial K5 + basal population (middle row panels, white arrows) following tamoxifen administration but retained in the K5-negative intermediate/superficial cell layers (middle row panels, yellow arrows) (Fig. 2B). As previously reported [17, 32, 34, 35], exposure of mice to BBN results in hyperplastic expansion of the K5 + basal cell population and near complete replacement of K5-negative intermediate/superficial cells with K5 + basal cells across the entire urothelium (Fig. 2B, bottom row, middle panel, black arrows). Importantly, tamoxifen administration to BBN-exposed GEMMs resulted in the replacement of Trim29 + luminal urothelium with a Trim29-negative K5 + basal population (Fig. 2B, bottom row, right panel, white arrows).

**Figure 3:**
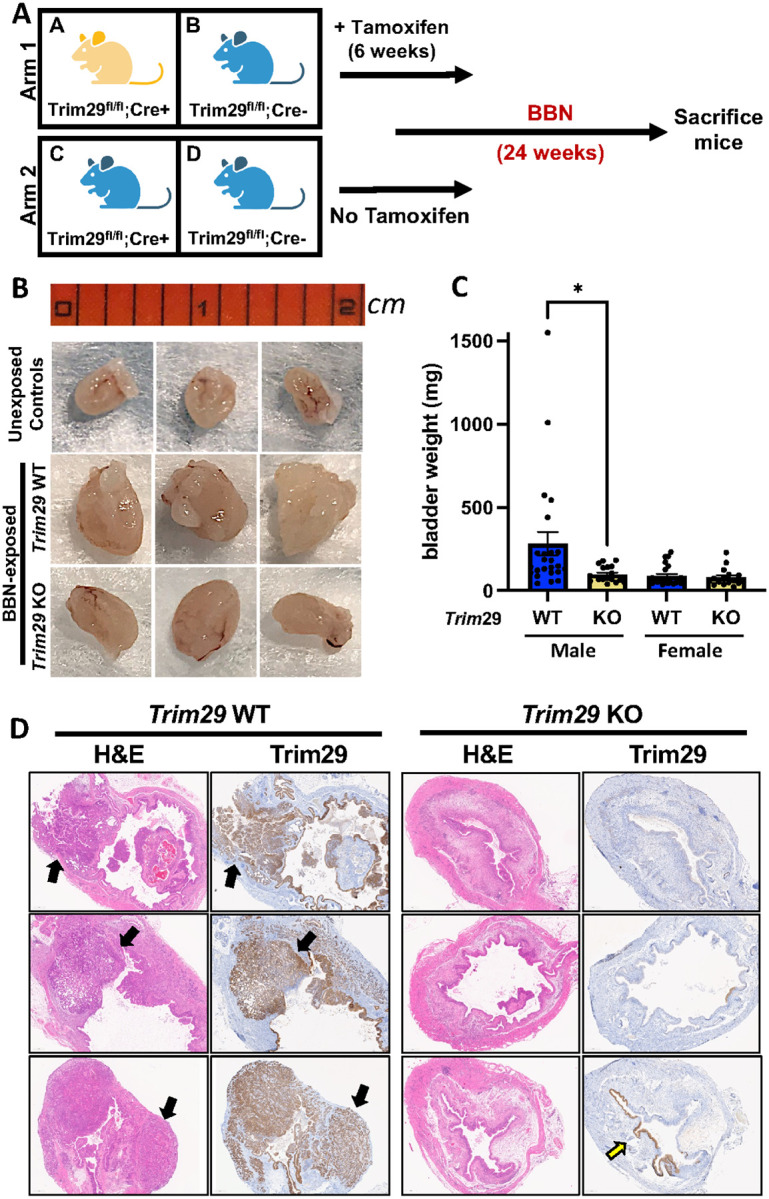
*Trim29* KO reduced BBN-induced bladder tumor burden. (A) Experimental schema. Arm 1 male and female mice (cohorts A&B) were administered tamoxifen chow for 3 weeks prior to initiation of 0.05% BBN in drinking water, which continued for 3 weeks following BBN initiation. Arm 2 mice (cohorts C&D) received a regular diet. *Trim29* WT control mice (cohorts B-D, represented by “blue” mouse) either did not receive tamoxifen chow or did not harbor a K5^CreERT2^ allele. *Trim29* KO mice (cohort A, “yellow” mouse) harbor a K5^CreERT2^ allele and received tamoxifen. (B) Representative bladder images of age matched *Trim29* WT (no BBN exposure), *Trim29* WT (+Tamoxifen, +BBN) and *Trim29* KO mice (+Tamoxifen, +BBN). (C) Bladder weights were significantly higher in the male *Trim29* WT mice (n = 24) as compared to *Trim29* KO (n = 18). * p <0.05 by student t test. (D) Representative histology and Trim29 IHC of tumors from BBN exposed *Trim29* WT and KO mice. *Trim29* KO mice display reduced tumor burden and reduced muscle-invasion as compared to the muscle-invasive tumors seen in *Trim29* WT mice (black arrows). Some *Trim29* KO mice display heterogeneous *Trim29* expression in urothelium (yellow arrow).

Trim29 KO in K5 + cells blocks bladder cancer formation and invasive progression. To determine the requirement for Trim29 in BBN-induced bladder cancer development, we generated four cohorts including both male (n = 42) and female (n = 39) Trim29^flox/flox^ mice, either harboring (cohorts A and C) or lacking (cohorts B and D) the K5^CreERT2^ allele. Mice in Arm 1 (cohorts A and B) received tamoxifen chow, whereas mice in Arm 2 (cohorts C and D) received regular chow (Fig. 3A). All cohorts were subsequently exposed to 0.05% BBN in drinking water for 24 weeks starting at 2–3 months of age. Cohort A (K5^CreERT2^; *Trim29*^flox/flox^, +tamoxifen) resulted in K5 + basal cell *Trim29* KO whereas cohorts B-D (Fig. 3A “blue” mice) retained Trim29 expression (*Trim29* WT) and controlled for effects of Cre or tamoxifen on bladder tumorigenesis. Additionally, age-matched littermate untreated controls were also evaluated. After 24 weeks of BBN exposure animals were sacrificed and bladders were examined.

Grossly, bladders from the *Trim29* WT controls (cohorts B-D) exposed to BBN showed enlarged nodular bladders, whereas the *Trim29* KO mice (cohort A) demonstrated smaller bladders with a smooth serosa similar to unexposed control mice (Fig. 3B). All three *Trim29* WT control cohorts (B-D) showed similar bladder weights (Sup. Figure 2A). Interestingly, the BBN-exposed bladders from *Trim29* WT male mice were larger than female *Trim29* WT bladders (Fig. 3C), reproducing the known male-female disparity in bladder cancer development [36, 37]. Notably, bladder weight was significantly reduced in the male *Trim29* KO mice (cohort A) as compared to *Trim29* WT (cohorts B-D) (Fig. 3C). To assess the tumor histology and invasiveness of the resulting bladder tumors in all cohorts, hematoxylin and eosin (H&E) staining and microscopic examination was performed. This analysis revealed that *Trim29* WT mice developed highly invasive, poorly differentiated tumors with extensive invasion into or beyond the muscularis propria (black arrows), whereas *Trim29* KO bladders displayed limited or no tumor burden (Fig. 3D). Notably, Trim29 was highly expressed in all tumors resulting from BBN exposure in the *Trim29* WT cohorts (Fig. 3D, Trim29 IHC). To confirm loss of Trim29 expression in the tamoxifen inducible KO (cohort A), all paraffin-embedded bladders were subjected to Trim29 IHC. Penetrance of the KO phenotype within individual male or female bladders ranged from complete KO (top two panels) to partial KO (yellow arrow, Fig. 3D). Nonetheless, 65% (22/34) of all male and female bladders within cohort A displayed > 90% loss of Trim29 expression in the urothelium.

**Figure 4:**
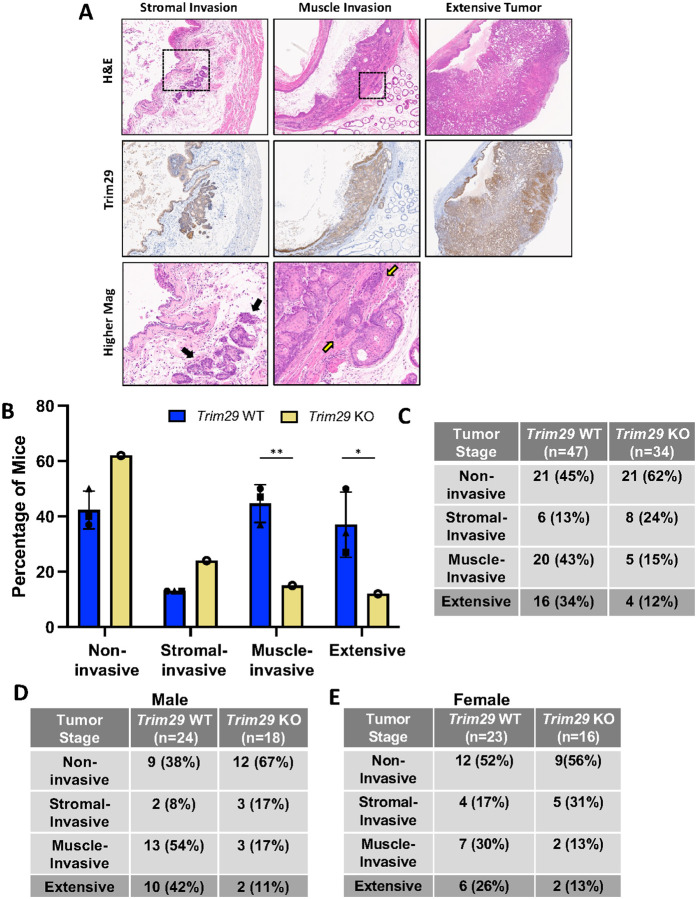
*Trim29* KO reduces BBN-induced bladder tumor muscle-invasion. (A) Histopathologic representation of T1 stromal invasive (black arrows), ≥T2 muscle-invasive (yellow arrows) and extensive tumors in *Trim29* WT mice. (B&C) Pathologic analysis of male and female mouse bladders from *Trim29* WT and KO mice demonstrated *Trim29* KO reduced percentage of mice with muscle-invasive and extensive tumors. *Trim29* WT groups (cohorts B-D) are indicated on the plot as follows: ▲ = cohort B (n = 24), ■ = cohort C (n = 15), ● = cohort D (n = 8). Trim29 KO (● = cohort A) n = 34. Statistical analysis using Fisher’s exact test was used comparing tumor stage distribution between both male and female *Trim29* KO (n = 34) and WT (n = 47) mice shows significantly lower frequency of muscle-invasive tumors in KO animals (15% vs. 43%, p = 0.0082). A similar trend was observed for extensive tumors (12% vs. 34%, p = 0.0353). Distribution of bladder tumor types between *Trim29* WT and KO tumors in (D) male and (E) female mice.

To quantitate the effect of *Trim29* KO on bladder tumor formation and invasive progression, H&E slides were examined by an expert genitourinary pathologist who was blinded to the genotype and cohort of the animals from which the bladders were derived. Each bladder was categorized for the presence or absence of stromal (T1, black arrows) or muscle-invasive tumor (≥ T2, yellow arrows) and for the presence or absence of extensive tumor, defined as a tumor which replaced > 50% of the bladder section (representative examples, Fig. 4A). Non-invasive tumor comprised of bladder tumor/dysplasia that displayed no evidence of local invasion into the basement membrane (Ta, CIS, atypia) was also examined. Animals were then grouped according to the most aggressive lesion seen in the bladder (i.e. a bladder exhibiting both non-invasive and muscle-invasive histology was placed in the muscle-invasive category). After histopathological classification, samples were then sorted by genotype and the presence or absence of tamoxifen administration. *Trim29* WT mice (cohorts B-D) were did not show any significant differences in histology or invasiveness related to tamoxifen exposure or Cre status (Sup. Figure 2B). These cohorts were thus grouped together as *Trim29* WT for subsequent analyses. Overall, *Trim29* KO bladders displayed > 60% reduction in the frequency of muscle-invasion and extensive tumor formation as compared to *Trim29* WT (Fig. 4B). Across male and female WT subgroups, MIBC developed in 43% of mice, including tumors which invaded through the muscle and into adjacent fat, while only 15% of *Trim29* KO mice developed MIBC (≥ T2) (Fig. 4B and 4C). Within the male mouse cohort, 54% of *Trim29* WT mice developed muscle-invasive (MI) bladder tumors (n = 13/24), whereas only 17% of KO mice (n = 3/18) developed MI tumors (Fig. 4D). Further, 42% of the *Trim29* WT male mice developed extensive tumors (n = 10/23), whereas only 11% of *Trim29* KO animals developed extensive MI tumors (n = 2/18). While the observed sex-based disparity in tumor development in response to BBN exposure is similar to current literature [38, 39], higher frequencies of advanced MI tumors were similarly observed in female WT mice (30%, n = 7/23) compared to KO mice (13%, n = 2/16) suggesting that the *Trim29* KO effect was not directly related to sex differences (Fig. 4E). Interestingly, 3 of the 5 muscle-invasive tumors and 3 of the 4 extensive tumors from *Trim29* KO mice retained Trim29 expression (Sup. Figure 3). This suggested that invasive tumors arising in the *Trim29* KO mice may have occurred due to incomplete urothelial KO. When we analyzed the frequency of MI and extensive tumor formation and excluded tumors that retained Trim29 expression in the KO cohort, the frequency of MIBC development was 6% and extensive tumor formation was 3%. These results demonstrate that TRIM29 expression in K5 + basal urothelial cells is critical for the development of muscle-invasive bladder tumors and that loss of TRIM29 blocks tumor formation and invasive progression regardless of sex-based differences in bladder tumor development.

**Figure 5:**
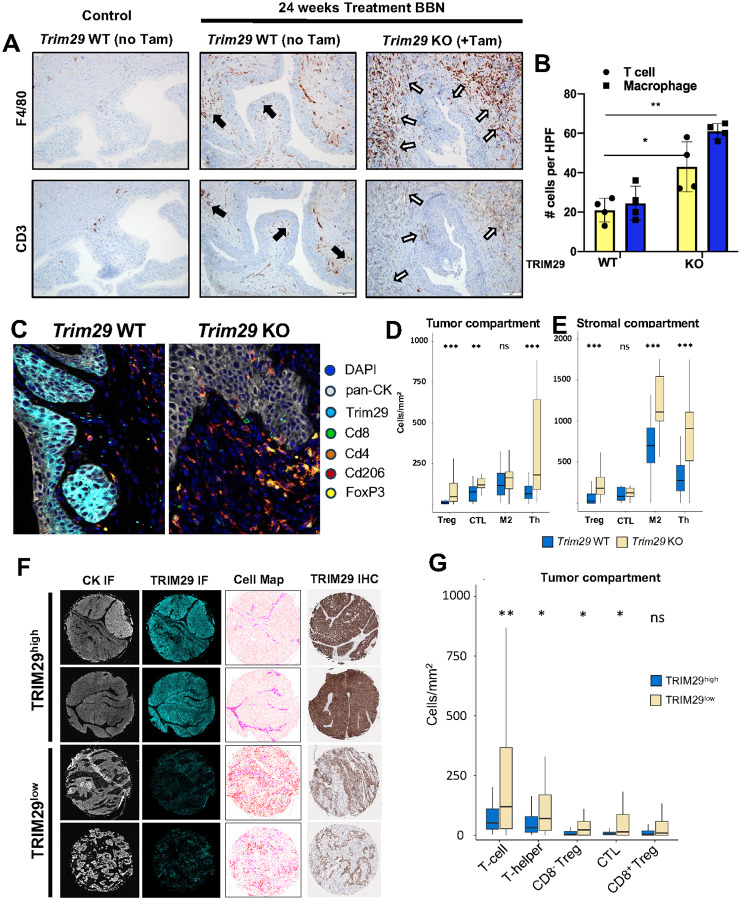
*Trim29* KO increases immune infiltration in the bladder. (A) IHC shows increased bladder infiltration with F4/80^+^ macrophages and CD3^+^ T cells in *Trim29* KO mice (black and white arrows). (B) Quantification of IHC high power fields (HPF). * p < 0.05; ** p < 0.01. (C) Representative ROIs of mIF staining of bladder tumors. Tissue immunotyping and quantification of immune cell densities (cells/mm^2^) in tumor epithelial (D) and stromal (E) compartments from *Trim29* WT and KO bladders. Immune cell subsets shown include CD4^+^FoxP3^+^ regulatory T cells (Treg), CD8^+^FoxP3^−^ cytotoxic T lymphocytes (CTL), CD206^+^ M2 macrophages (M2), and CD4^+^FoxP3^−^ helper T cells (Th). Statistical comparisons were performed using the Wilcoxon rank-sum test for each cell type within each compartment. For the tumor compartment (D), p-values were: Treg = 4.9×10^−4^, CTL = 9.3×10^−3^, M2 = 0.16, Th = 5.5×10^−5^. For the stromal compartment (E), p-values were: Treg = 2.4×10^−5^, CTL = 0.3, M2 = 2.4×10^−4^, Th = 1.8×10^−5^. (F) Representative mIF and IHC images of cores from human bladder cancer TMA BL2081c (mIF antibody panel H1). Cores were classified as TRIM29^high^ or TRIM29^low^ based on the percent of cytokeratin-positive (CK^+^) epithelial cells that co-stained for TRIM29 in the total core from inform datasets: (CK^+^/TRIM29^+^)/(CK^+^) × 100. Cores with >50% TRIM29 co-staining were grouped TRIM29^high^ (n = 84); all others were grouped TRIM29^low^ (n = 49). TRIM29 status was confirmed by TRIM29 IHC. Cell types were assigned based on mIF phenotype including T cells (CD3^+^), T-helper (CD3^+^CD8^−^), CD8^−^ Treg (CD3^+^CD8^−^FOXP3^+^), CTL (CD8^+^), and CD8^+^Treg (CD8^+^FOXP3^+^) (G) TRIM29^low^ tumors have significantly increased T cells, T helper, Treg and CTL as compared to TRIM29^high^ bladder tumors. Immune-cell densities (cells/mm^2^) were compared between TRIM29^high^ and TRIM29^low^ cores using two-sided Wilcoxon rank-sum tests.

Loss of TRIM29 enhances immune cell recruitment to the bladder tumor microenvironment. TRIM29 is an emerging negative regulator of innate immune signaling with a purported role in suppressing inflammatory signaling and immune cell recruitment [21–24]. We found that urothelial Trim29 expression correlated with carcinogen-induced bladder inflammation (Fig. 1D, Sup. Figure 1). We therefore hypothesized that TRIM29 expression in the tumor would suppress immune recruitment to the bladder TME. To measure this, we quantitated F4/80^+^ macrophages and CD3^+^ T cells in the bladder following chronic BBN exposure using IHC. BBN-exposed *Trim29* WT bladders exhibited modest immune infiltration, with scattered macrophages and T cells (Fig. 5A, black arrows). In contrast, *Trim29* KO bladders displayed significantly increased immune cell recruitment to the bladder, with over a two-fold increase in both F4/80^+^ macrophages and CD3^+^ T cells, suggesting a more robust immune response in the absence of Trim29 (Fig. 5A, white arrows and Fig. 5B).

Previous reports have detailed the negative influence of epithelial Trim29 expression on immune cell recruitment in response to viral infection in the intestinal tract [24]. To probe the influence of tumor intrinsic Trim29 expression on the bladder immune TME, we performed spatially resolved multiplex immunofluorescence (mIF) analysis and quantitative immunotyping to assess immune cell infiltration on tumors from *Trim29* KO and WT mice exposed to BBN. Tumors were stained for pan-CK (epithelial marker), CD8 (cytotoxic T cells), CD4 (helper T cells), CD206 (M2 macrophages), FoxP3 (regulatory T cells), and Trim29 (Fig. 5C). Tissues were segmented into tumor vs stroma compartments using machine learning (Akoya Biosciences inForm software). While *Trim29* WT tumors were largely immune-excluded, *Trim29* KO tumors exhibited a significant increase in tumor-infiltrating helper T cells (Th), cytotoxic T lymphocytes (CTLs), and FoxP3^+^ regulatory T cells (Tregs) (Fig. 5D). Similarly, the stromal compartment showed elevated densities of peritumoral Th cells, M2-like macrophages, and Tregs in *Trim29* KO mice, suggesting that tumoral expression of Trim29 influenced immune recruitment to both the tumor and surrounding stroma (Fig. 5E). Interestingly, in one *Trim29* KO mouse, a muscle-invasive Trim29^+^ tumor (Sup. Figure 4A&B, black arrows) emerged alongside a neighboring Trim29^−^ tumor which was not muscle-invasive (Sup. Figure 4A&B, white arrows). mIF profiling of the Trim29^−^ tumor demonstrated a dense infiltration of immune cells, in contrast to the adjacent muscle-invasive Trim29^+^ tumor, which was largely devoid of immune infiltration (Sup. Figure 4D-F). These results suggest that Trim29 expression in the urothelium and tumor reduces infiltrating immune populations to modulate the bladder immune TME during tumorigenesis.

We next asked whether the relationship between TRIM29 expression and immune infiltration observed in mice translated to human bladder tumors. To assess this, a human bladder cancer TMA (BL2081c) consisting of 191 tumor cores was stained, segmented, and phenotyped using mIF analysis (Sup. Table 4, antibody panel H1). Tumor in each core was identified by cytokeratin staining (CK^+^). Each tumor core was classified as TRIM29^high^ or TRIM29^low^ based on the percentage of CK^+^ epithelial cells that positively co-stain for TRIM29 (≥ 50%) (Fig. 5F). TRIM29 expression was also confirmed with IHC. We then compared densities of various immune cells within TRIM29^high^ and TRIM29^low^ tumor cores. TRIM29^low^ tumors showed higher densities of total CD3^+^ T cells, CD3^+^CD8^−^ helper T cells, CD3^+^CD8^−^ FOXP3^+^ Tregs, and CD8^+^ CTLs relative to TRIM29^high^ tumor cores (Fig. 5G). We next assessed two deidentified individual human MIBCs by mIF using human antibody panel H2 (Sup. Table 4). Sample 1, which was TRIM29^low^, displayed significantly more CD8^+^ T cells, CD4^+^ Th cells, and M2-macrophages compared to sample 2 which was TRIM29^high^ (Sup. Figure 5). These results suggest that TRIM29 expression in human bladder tumors is linked to reduced lymphoid infiltration and a more immune-excluded microenvironment similar to the GEMM model.

TRIM29 expression is associated with loss of STING in the urothelium and tumor. TRIM29 has been implicated in the negative regulation of STING, MAVS, and NEMO, three central adaptor protein nodes in cytoplasmic nucleic acid sensing and subsequent inflammatory phospho-relay [20–23, 40]. In immune cells, activation of cytoplasmic nucleic acid sensing induces upregulation of TRIM29 resulting in ubiquitination and decreased levels of STING. We postulated that TRIM29 upregulation in the urothelium in response to chronic inflammation (Fig. 1A) or BBN (Fig. 1D), might also downregulate the expression of STING to remodel the bladder immune TME.

**Figure 6:**
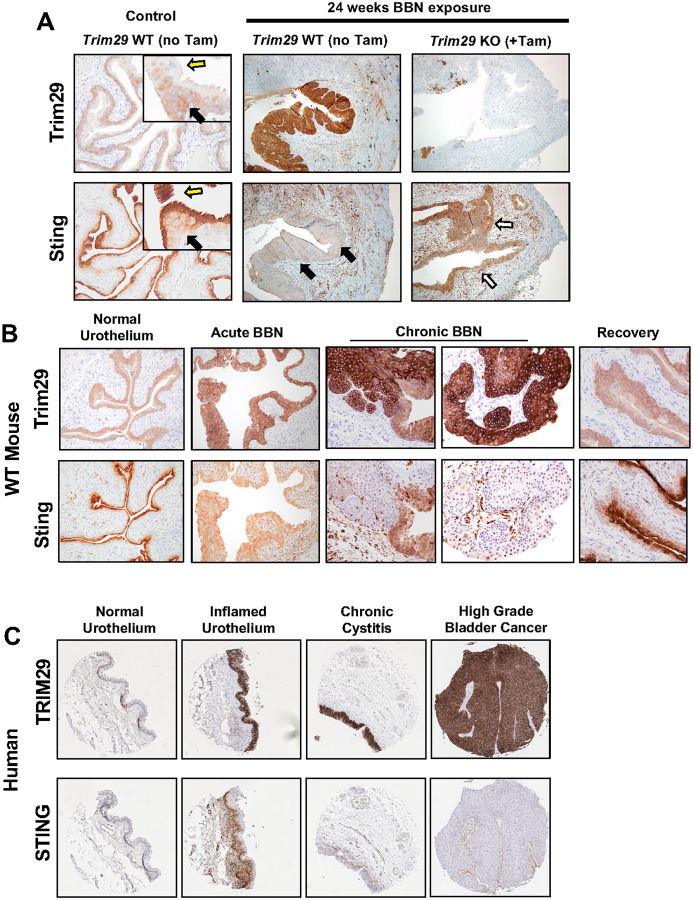
Inflammation upregulates TRIM29 in the urothelium and is associated with low STING expression. (A) Trim29 and Sting expression in normal control and BBN exposed bladders. Trim29 increases after BBN exposure, but Sting levels only increase after *Trim29* KO. (B) BBN exposure leads to increased Trim29 and Sting protein levels in the urothelium at 2 weeks (acute BBN) followed by reduced Sting protein at 16 weeks (chronic BBN). 18 weeks after withdrawal from acute BBN exposure, Trim29 and Sting return to baseline. (C) TRIM29 and STING IHC in human normal, inflamed, chronic cystitis and bladder cancer.

To determine whether Trim29 is associated with Sting abundance *in vivo*, we evaluated Sting expression in bladder tissues from *Trim29* WT and *Trim29* KO mice following 24 weeks of BBN exposure (Fig. 6A). In unexposed WT bladders, Trim29 is moderately expressed in the basal urothelium (black arrow) and less in terminally differentiated superficial cells (yellow arrow). In contrast, Sting IHC shows an inverse pattern and is significantly upregulated in luminal superficial cells (yellow arrow) and low in the intermediate/basal urothelial layer (black arrow) (Fig. 6A). However, following 24 weeks BBN exposure, Trim29 was highly upregulated across the urothelium, whereas Sting expression remained low in urothelial cells (black arrow) (Fig. 6A). In contrast, Sting expression is significantly elevated in BBNexposed *Trim29* KO bladders (white arrows). To further demonstrate that Sting expression was linked to Trim29, we stained the bladder with discrepant Trim29^+^ and Trim29^−^ tumors for Sting (Sup. Figure 4B). We found that while the Trim29^−^ tumor displayed relatively high Sting expression, the muscle-invasive Trim29^+^ tumor had low Sting expression, particularly within the core of the tumor (Sup. Figure 4C).

To better understand the dynamics of Trim29 and Sting expression in the bladder during inflammatory stress, C57BL/6J mice were exposed to BBN acutely (2 and 4 weeks) and chronically (12 and 16 weeks) and Trim29 and Sting were examined by IHC. Interestingly, while Trim29 upregulation occurred early and persisted throughout BBN exposure, Sting expression increased in the basal urothelium following acute exposure before being suppressed at later time points (Fig. 6B). To assess whether these changes in Trim29 and Sting expression persist after resolution of inflammation, we analyzed bladder tissue after an 18-week recovery period following acute BBN exposure. These bladders demonstrate a return to unexposed quiescent baseline expression levels, indicating that Trim29 upregulation and Sting suppression is a transient response to acute BBN exposure.

To examine whether a similar dynamic might occur in human bladders, TRIM29 and STING were examined in normal urothelium, inflamed urothelium, chronic cystitis and tumor. As in the mouse model, TRIM29 and STING are relatively low in normal urothelium, but both increase with acute inflammation. However, the persistently high levels of TRIM29 in chronic cystitis and tumor were associated with suppressed STING expression (Fig. 6C). Taken together, these results suggest that TRIM29 upregulation is an acute and long-term response to inflammation that is associated with suppressed STING levels during sustained inflammatory conditions.

**Figure 7:**
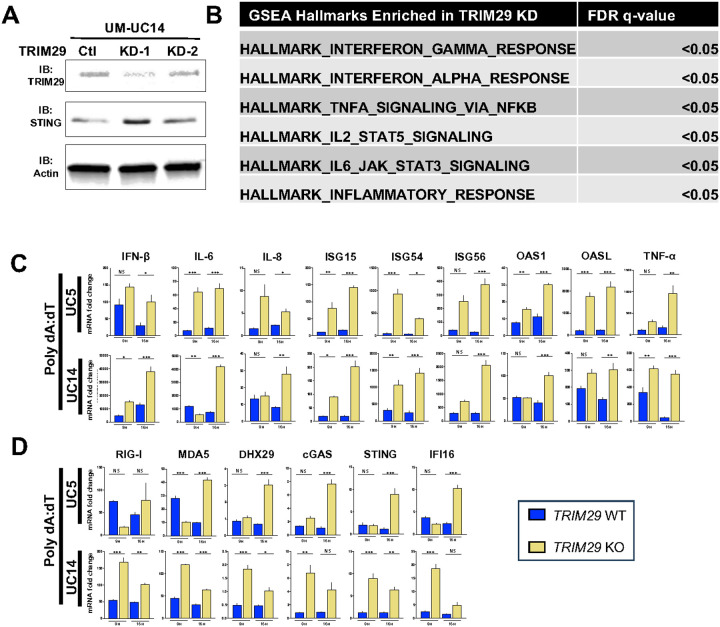
Decreasing TRIM29 expression in bladder cancer cells enhances STING signaling. (A) TRIM29 KD in bladder cancer cells is associated with increased STING protein levels. (B) TRIM29 KD in UM-UC14 cells results in decreased expression of multiple GSEA hallmarks related to immune signaling as measured by Bru-Seq. (C&D) qRT-PCR analysis of innate immune gene expression in UM-UC5 and UM-UC14 *TRIM29* WT (blue) and KO (yellow) cells transfected with poly(dA:dT). Cells were harvested at 9 or 16 hours post-stimulation for RNA extraction. Expression was measured by RT-qPCR for cytokines and interferon-stimulated genes (C) and innate immune sensors (D) then quantified relative to GAPDH using the ΔΔCt method. Bars represent mean ± SEM from technical triplicates. Statistical comparisons between WT and KO were performed using one-way ANOVA to determine overall statistical significance and pairwise comparisons were conducted using Bonferroni-corrected t-tests. ns = not statistically significant; * p < 0.05; ** p < 0.01; *** p < 0.001. Results were considered statistically significant at p < 0.05.

TRIM29 loss results in STING upregulation and downstream STING target gene upregulation. Our *Trim29* KO model suggested that TRIM29 regulates STING at the protein level. To examine if this was true *in vitro*, we knocked down TRIM29 in UM-UC14 bladder cancer cell lines with two independent siRNAs. We found that TRIM29 knockdown (KD) was inversely correlated with proportional increases in STING protein levels (Fig. 7A).

BBN-induced tumors exhibit genomic instability, a hallmark of most cancers [33]. dsDNA released from tumor cells can be sensed by the STING pathway in both epithelial and immune cells to facilitate antitumor immune surveillance, and STING signaling is required for generating tumor-specific T-cell responses [41]. STING activation triggers IRF3 phosphorylation, dimerization, and nuclear translocation, where it drives the transcription of type-I IFN and numerous interferon-stimulated genes (ISGs) as part of the innate immune response [42, 43]. To determine whether TRIM29 negatively regulates the innate immune response in tumors, we performed nascent transcriptomic profiling (Bru-seq) in UM-UC14 human bladder cancer cells following TRIM29 KD. TRIM29 KD induced the upregulation of interferon-stimulated genes (ISGs), inflammatory mediators, and innate immune sensors compared with control cells (Sup. Figure 6). Among the most strongly induced transcripts were IFIT1–3, OASL, HERC, and IFI44, which are canonical targets of type-I IFN signaling, in addition to pro-inflammatory cytokines such as IL8 and adaptors of the RIG-I/MDA-5 signaling axis. Notably, several of these genes have been shown to be directly regulated by STING activity. Gene set enrichment analysis (GSEA) revealed significant upregulation of several inflammatory and immune signaling pathways in TRIM29-deficient cells, including IFN-α and IFN-γ responses, TNFα signaling via NF-κB, IL2-STAT5 and IL6-STAT3 pathways, and a global inflammatory response hallmark (FDR q < 0.05 for all; Fig. 7B). These findings further support the role of TRIM29 as a negative regulator of innate immune sensing pathways in bladder cancer cells and suggest that its loss may enrich STING signaling, leading to the induction of pro-inflammatory cytokines and immune cell recruitment.

STING pathway activation is induced via the direct sensing of damage associated molecular patterns (DAMP) such as intracytoplasmic dsDNA by cyclic GMP-AMP synthase (cGAS) and triggers the production of inflammatory cytokines [44, 45]. STING activation can be artificially stimulated by transfection with synthetic dsDNA such as poly(dA:dT) or interferon stimulatory DNA (ISD) [27]. To assess whether TRIM29 suppresses STING target gene mRNA expression following nucleic acid stimulation in bladder cancer cells, we treated *TRIM29* WT and KO UM-UC5 and UM-UC14 bladder cancer cells with poly(dA:dT) or ISD and measured the induction of target gene expression by RT-qPCR at 9 and 16 hours post-stimulation. In both bladder cancer lines, stimulation with poly(dA:dT) in *TRIM29* KO significantly upregulated transcription of key cytokines, chemokines, and antiviral effectors compared to *TRIM29* WT (Fig. 7C). Notably, transcripts of nucleic acid sensors and innate immune adaptors (MDA5, RIG-I, cGAS, and STING) were significantly more induced in *TRIM29* KO cells (Fig. 7D). This was generally consistent across both early (9 hr) and late (16 hr) timepoints, highlighting sustained enhancement of positive feedback in response to innate immune signaling in the absence of TRIM29. Similar findings were observed for ISD stimulation in *TRIM29* WT and KO cells (Sup. Figure 7). These findings further support a model in which TRIM29 functions as a molecular brake on DAMP activation of innate immunity in tumor cells, suppressing STING-mediated transcriptional responses that would otherwise activate pro-inflammatory signaling during invasive progression.

## Discussion

We have previously shown that TRIM29 is highly expressed in MIBC and upregulated as part of a TP63 regulated gene program in cancer [11]. We have also shown that overexpression of TRIM29 is sufficient to induce tumorigenesis and MIBC development in a GEMM [10]. However, prior to this study it was not clear what signals induce TRIM29 upregulation in the bladder urothelium and whether TRIM29 was required for efficient tumor formation and progression. Likewise, recent publications have shown an important role for TRIM29 in modulation of the immune microenvironment during viral infection [24], but whether this occurred in bladder cancer was unclear. In this study, we demonstrate that TRIM29 is dynamically upregulated in response to inflammatory stress, sustained in chronically inflamed urothelium, and is critical to the formation and progression of MIBC. We find that TRIM29 expression is associated with reduced immune cell recruitment to the bladder TME, likely in part due to suppression of innate immune signaling in bladder cancer cells. Together, these findings suggest that TRIM29 contributes to bladder tumorigenesis not only by driving basal cell migration, but also by mediating an epithelial-driven immunosuppressive response that alters the bladder immune TME to permit tumor development and invasion.

In the quiescent bladder, the innermost urothelial compartment harbors two distinct basal cell populations: K5 basal cells that line the basement membrane, and a sparse population of K14 cells speculated to be the stem-like population that gives rise to all cells in the urothelium [4]. Upon urothelial insult by infection, carcinogen exposure, or mechanical stress, terminally differentiated epithelial cells (K20+) are lost and a regenerative program induces basal stem cells (K5 + K14+) to re-enter the cell cycle to replenish lost cells and repair tissue. Our results support a model in which the TP63-TRIM29-KRT14 axis, an established driver of basal tumor identity [11, 12], is activated in response to stress as part of this regenerative program in basal cells. TRIM29 expression is an acute and chronic response to inflammation and urothelial damage. In human tissues, TRIM29 expression was markedly elevated in chronic cystitis and highly expressed across progressive stages of bladder cancer. In the BBN carcinogenesis model, Trim29 was rapidly induced during acute inflammatory remodeling and remained elevated with chronic carcinogen exposure. Trim29 upregulation coincided with expansion of basal K5 and K14 cell populations, suggesting that TRIM29 may also play a role bladder tissue wound repair and the regeneration of urothelial lineages.

By conditionally deleting *Trim29* in the K5 + urothelium, we demonstrate that basal cell-specific Trim29 expression is crucial for BBN-induced MIBC formation and progression. While *Trim29* WT control mice developed extensive, high-grade, muscle-invasive tumors, *Trim29* KO animals displayed dramatically reduced tumor burden, reduced frequencies of muscle-invasion, and overall impaired progression. These findings are consistent with our prior studies demonstrating that TRIM29 overexpression is sufficient to drive MIBC, is enriched in basal tumors, and directly regulates invasive behavior [10–12]. Notably, the limited number of muscle-invasive tumors that did form in the *Trim29* KO cohort often retained Trim29 expression, suggesting that even the low frequency of invasive tumor progression observed in the *Trim29* KO group were still dependent on Trim29. These findings provide functional evidence that TRIM29 is a critical mediator of bladder tumor formation and invasive progression *in vivo*.

Beyond its association with bladder tumor invasion, our data establish TRIM29 as a potent negative regulator of the urothelial immune microenvironment. DAMPs, including cytosolic dsDNA, activate and upregulate STING and other innate immune adapters to induce the production of type I IFN and pro-inflammatory cytokines that recruit immune cells to the bladder and trigger apoptosis of infected or damaged epithelial cells [44]. Terminally differentiated superficial cells are luminal facing and form an environmentally exposed barrier to pathogens or genotoxic agents in urine and are thus tasked with front line innate immune detection. We find that TRIM29 expression is low whereas STING is highly expressed in the superficial cell layer in the bladder during homeostasis, while the inverse is observed in the basal compartment. Following BBN exposure *Trim29* WT mouse bladders experience a loss of the superficial Trim29^−^/Sting^+^ cells, which are replaced with Trim29^+^/Sting^−^ cells across the urothelial expanse. This loss of Sting expression in the urothelium, perhaps through Trim29-mediated silencing, allows urothelial cell survival in the face of ongoing stress to maintain the tissue integrity of the bladder, but also increases the risk of tumor development by facilitating the survival of damaged cells.

In other systems, TRIM29-mediated negative regulation of innate immune adaptors also downregulates immune activation and recruitment, likely to prevent an overcommitted immune response [20–24]. Here we show that loss of Trim29 in the epithelial compartment of the bladder is associated with dramatic reductions in infiltration of immune cells to the bladder TME. This data suggests a TRIM29 regulatory pathway, wherein damaged or inflamed urothelial tissue continues to regenerate and expand but loses immune surveillance capacity. The consequences of this shift may be that the urothelium can regenerate, but becomes increasingly immune-excluded, allowing the accumulation of mutation, immune evasion, and ultimately invasive progression in the presence of genotoxic stress.

In both mouse and human tissues, loss of TRIM29 expression was associated with upregulated IFN-stimulated genes, pro-inflammatory cytokines, and nucleic acid sensors, suggesting upregulation of antiviral and inflammatory programs. Functional assays confirmed that TRIM29 loss sensitized bladder tumor cells to nucleic acid stimulation, resulting in robust cytokine induction. These findings support a model in which TRIM29 acts as a molecular brake on inflammation, suppressing immune cell recruitment and fostering an immune-excluded TME conducive to tumor progression.

This work also suggests areas for future investigation. While we establish an inverse association between TRIM29 and STING in the urothelium and tumors, TRIM29 has been shown to negatively regulate multiple immune adaptors such as MAVS and NEMO, and critical inflammasomes in the intestinal tract. While our data suggest STING as a target of TRIM29 in the bladder, it is likely that TRIM29-mediated immune suppression involves multiple innate immune adapters in the bladder. In addition, while our data implicate TRIM29 in immune exclusion, the functional impact of the individual immune cell populations enriched in *Trim29* KO tumors is unclear. Future work to examine the functional role of individual immune cell types will be critical to understand how the bladder immune TME regulates tumorigenesis.

This work also suggests that targeting TRIM29 directly could restore immune visibility of TRIM29^+^ bladder tumors and enhance responsiveness to immunotherapies. Although TRIM29 lacks easily druggable enzymatic domains, its ubiquitin ligase activity and protein-protein interactions may be amenable to pharmacologic inhibition or targeted protein degradation strategies [46]. Moreover, TRIM29 expression may serve as a biomarker to stratify patients likely to exhibit poor responses to immunotherapy.

In summary, we demonstrate that TRIM29 is upregulated by inflammatory stress, required for MIBC initiation and progression from basal urothelial cells, and functions as a suppressor of innate immune signaling. These findings implicate TRIM29 as a molecular hub linking inflammation, lineage specification, and immune evasion in bladder cancer. By integrating urothelial and immune biology, our work highlights TRIM29 as both a biomarker of aggressive basal tumors and a potential therapeutic target to restore anti-tumor immunity.

## Supplementary Material

Supplementary Files

This is a list of supplementary files associated with this preprint. Click to download.

- SuppTables1626.docx
- SupplementalFigurelegends.docx
- Trim29KOFigures26269.png
- Trim29KOFigures262610.png
- Trim29KOFigures262611.png
- Trim29KOFigures262612.png
- Trim29KOFigures262613.png
- Trim29KOFigures262614.png
- Trim29KOFigures262615.png
- Trim29KOFigures26261.png

## Statement of Significance

Understanding the TRIM29-mediated molecular mechanisms that drive bladder cancer development, invasive progression, and immune response silencing will enable us to develop better therapies to prevent invasive progression and sensitize tumors to immunotherapy.
